# Supplementary material for: Multi‐dataset identification of innovative feature genes and molecular mechanisms in keratoconus
Source: J Cell Mol Med. 2024 Sep 19;28(18):e70079. doi: 10.1111/jcmm.70079 (PMC11412914; doi:10.1111/jcmm.70079)
Supplement: Supplementary file 2 — Material S2. [file JCMM-28-e70079-s002.zip › 7.GSEA analysis/3.DBNDD1/KEGG_GLYCOSYLPHOSPHATIDYLINOSITOL_GPI_ANCHOR_BIOSYNTHESIS.html]

Details for gene set KEGG\_GLYCOSYLPHOSPHATIDYLINOSITOL\_GPI\_ANCHOR\_BIOSYNTHESIS[GSEA]

|  || Dataset | DBNDD1.group.cls#h\_versus\_l |
| Phenotype | group.cls#h\_versus\_l |
| Upregulated in class | h |
| GeneSet | KEGG\_GLYCOSYLPHOSPHATIDYLINOSITOL\_GPI\_ANCHOR\_BIOSYNTHESIS |
| Enrichment Score (ES) | 0.19406995 |
| Normalized Enrichment Score (NES) | 0.5950588 |
| Nominal p-value | 0.87903225 |
| FDR q-value | 1.0 |
| FWER p-Value | 1.0 |
Table: GSEA Results Summary

  

Fig 1: Enrichment plot: KEGG\_GLYCOSYLPHOSPHATIDYLINOSITOL\_GPI\_ANCHOR\_BIOSYNTHESIS      
 Profile of the Running ES Score & Positions of GeneSet Members on the Rank Ordered List

  

| SYMBOL | TITLE | RANK IN GENE LIST | RANK METRIC SCORE | RUNNING ES | CORE ENRICHMENT || 1 | PGAP1 | na | 5326 | 0.169 | -0.0173 | Yes |
| 2 | PIGW | na | 6722 | 0.131 | 0.0477 | Yes |
| 3 | GPAA1 | na | 10100 | 0.070 | 0.0068 | Yes |
| 4 | PIGL | na | 10375 | 0.066 | 0.0521 | Yes |
| 5 | PIGY | na | 10725 | 0.062 | 0.0916 | Yes |
| 6 | PIGP | na | 11953 | 0.048 | 0.0945 | Yes |
| 7 | PIGK | na | 12908 | 0.039 | 0.0984 | Yes |
| 8 | PIGB | na | 13149 | 0.037 | 0.1212 | Yes |
| 9 | PIGU | na | 15251 | 0.021 | 0.0775 | Yes |
| 10 | PIGS | na | 15374 | 0.020 | 0.0902 | Yes |
| 11 | PIGH | na | 16788 | 0.011 | 0.0581 | Yes |
| 12 | PIGF | na | 17352 | 0.007 | 0.0477 | Yes |
| 13 | PIGG | na | 18075 | 0.002 | 0.0285 | Yes |
| 14 | PIGN | na | 18551 | -0.001 | 0.0159 | Yes |
| 15 | PIGO | na | 19397 | -0.008 | -0.0023 | Yes |
| 16 | PIGM | na | 19531 | -0.009 | 0.0009 | Yes |
| 17 | PIGQ | na | 20787 | -0.020 | -0.0192 | Yes |
| 18 | PIGX | na | 20885 | -0.021 | -0.0051 | Yes |
| 19 | PIGV | na | 21534 | -0.027 | -0.0018 | Yes |
| 20 | DPM2 | na | 22640 | -0.039 | -0.0024 | Yes |
| 21 | PIGT | na | 22903 | -0.042 | 0.0237 | Yes |
| 22 | PIGZ | na | 24652 | -0.066 | 0.0261 | Yes |
| 23 | PIGC | na | 26539 | -0.098 | 0.0508 | Yes |
| 24 | GPLD1 | na | 26579 | -0.099 | 0.1294 | Yes |
| 25 | PIGA | na | 27993 | -0.131 | 0.1941 | Yes |
Table: GSEA details [plain text format]

  

Fig 2: KEGG\_GLYCOSYLPHOSPHATIDYLINOSITOL\_GPI\_ANCHOR\_BIOSYNTHESIS      
 Blue-Pink O' Gram in the Space of the Analyzed GeneSet

  

Fig 3: KEGG\_GLYCOSYLPHOSPHATIDYLINOSITOL\_GPI\_ANCHOR\_BIOSYNTHESIS: Random ES distribution      
 Gene set null distribution of ES for **KEGG\_GLYCOSYLPHOSPHATIDYLINOSITOL\_GPI\_ANCHOR\_BIOSYNTHESIS**
